# Estimating Changes in Facility Methicillin-Resistant *Staphylococcus aureus* (MRSA) Infection Rates Due to Changes in MRSA Precaution Policy

**DOI:** 10.1093/cid/ciag176

**Published:** 2026-03-13

**Authors:** Karim Khader, Candace Haroldsen, Vanessa Stevens, Lindsay Visnovsky, Martin Evans, Loretta Simbartl, Brian McCauley, Matthew Samore, Michael Rubin

**Affiliations:** Informatics, Decision Enhancement, and Analytical Sciences (IDEAS) Center, VA Salt Lake City Health Care System, Salt Lake City, Utah, USA; Division of Epidemiology, Department of Internal Medicine, University of Utah School of Medicine, Salt Lake City, Utah, USA; Informatics, Decision Enhancement, and Analytical Sciences (IDEAS) Center, VA Salt Lake City Health Care System, Salt Lake City, Utah, USA; Division of Epidemiology, Department of Internal Medicine, University of Utah School of Medicine, Salt Lake City, Utah, USA; Informatics, Decision Enhancement, and Analytical Sciences (IDEAS) Center, VA Salt Lake City Health Care System, Salt Lake City, Utah, USA; Division of Epidemiology, Department of Internal Medicine, University of Utah School of Medicine, Salt Lake City, Utah, USA; Informatics, Decision Enhancement, and Analytical Sciences (IDEAS) Center, VA Salt Lake City Health Care System, Salt Lake City, Utah, USA; Division of Epidemiology, Department of Internal Medicine, University of Utah School of Medicine, Salt Lake City, Utah, USA; National Infectious Diseases Service, Specialty Care Services, Veterans Health Administration, US Department of Veterans Affairs, Washington, District of Columbia, USA; National Infectious Diseases Service, Specialty Care Services, Veterans Health Administration, US Department of Veterans Affairs, Washington, District of Columbia, USA; National Infectious Diseases Service, Specialty Care Services, Veterans Health Administration, US Department of Veterans Affairs, Washington, District of Columbia, USA; Informatics, Decision Enhancement, and Analytical Sciences (IDEAS) Center, VA Salt Lake City Health Care System, Salt Lake City, Utah, USA; Division of Epidemiology, Department of Internal Medicine, University of Utah School of Medicine, Salt Lake City, Utah, USA; Informatics, Decision Enhancement, and Analytical Sciences (IDEAS) Center, VA Salt Lake City Health Care System, Salt Lake City, Utah, USA; Division of Epidemiology, Department of Internal Medicine, University of Utah School of Medicine, Salt Lake City, Utah, USA

**Keywords:** contact precautions, MRSA prevention, infection control, hospital epidemiology

## Abstract

**Background:**

The effectiveness of contact precautions (CP) and active surveillance (AS) for preventing methicillin-resistant *Staphylococcus aureus* (MRSA) in acute care remains uncertain. Some studies suggest CP reduces MRSA spread, while others report limited benefit. The COVID-19 pandemic disrupted MRSA prevention practices in the VA, creating an opportunity to assess their impact on MRSA healthcare-associated infections (HAIs).

**Methods:**

We studied 121 VA acute care hospitals from July 2020–June 2022. Facility practices (AS, CP for colonized [CPC], CP for infected [CPI]) were assessed via national surveys. Patient-level data identified MRSA HAIs (incident cultures ≥3 days postadmission). Secondary outcomes included sterile-site infections and 30-day postdischarge cultures. Associations between practices and HAI rates were estimated using Poisson, negative binomial, and mixed-effects Poisson regression, adjusting for baseline MRSA burden, COVID-19 admissions, culturing intensity, and hospital characteristics.

**Results:**

Among 905 164 admissions, 1708 incident MRSA cultures were identified. Many facilities suspended at least one prevention practice early in the pandemic, though most later reinstated them. In simpler models, discontinuation of AS, CPC, or CPI was associated with higher MRSA rates, but these associations were attenuated after adjustment for baseline burden. Mixed-effects models found no significant associations, and results were consistent across secondary outcomes.

**Conclusions:**

Discontinuation of MRSA prevention practices during the pandemic was not consistently linked to increased HAIs after accounting for baseline burden. Findings emphasize the role of facility-specific factors and modeling assumptions in evaluating infection control. Unmeasured pandemic-related practices (eg, masking, PPE use) likely also influenced transmission, highlighting the need for flexible, context-sensitive, evidence-based infection prevention policies.

## Teaser

This study leveraged COVID-19–related changes in MRSA prevention across 121 VA hospitals to assess effects of contact precautions and surveillance. Effects varied by model and baseline burden, underscoring facility-specific factors and methodological choices in infection control evaluations.

## Graphical Abstract

**Graphical Abstract:**
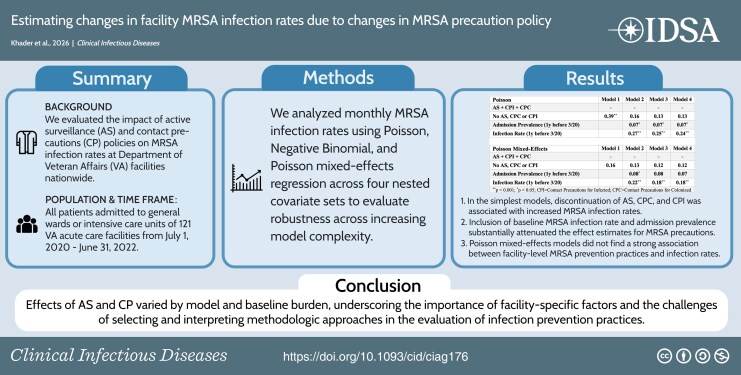
This graphical abstract is also available at Tidbit: https://tidbitapp.io/institutional-portal/clinical-infectious-diseases/tidbits/estimating-changes-in-facility-mrsa-infection-rates-due-to-changes-in-mrsa-precaution-policy-d080e477-cd24-4080-90aa-cfcec3effb34/update

The extent to which contact precautions (CP) alone and in combination with active surveillance (AS) contribute to preventing transmission of endemic multidrug-resistant organisms (MDRO) in acute care settings remains unsettled. Several studies in the past few years have challenged the effectiveness of using CP for preventing the spread of methicillin-resistant *Staphylococcus aureus* (MRSA) [1–3]. Other studies support the effectiveness of CP in preventing the spread of MRSA in acute care [4–6], including our previous work which estimated a reduction in transmissibility of MRSA from patients on CP by approximately half [7].

Starting in 2007, the Veterans Affairs (VA) Healthcare System implemented the MRSA Prevention Initiative [8] which included AS for all patients admitted to acute care hospital wards at hospital admission, discharge, and ward transfer, along with CPs for MRSA-colonized patients (CPC) and identified MRSA-infected patients (CPI). The COVID-19 pandemic significantly disrupted routine infection prevention practices in healthcare settings and accelerated an existing trend toward discontinuing or reducing the intensity of these infection prevention practices [9, 10]. In March 2020, VA policy was modified to enable VA Acute Care Medical Centers (VAMCs) to suspend AS for MRSA, CPC, or CPI, individually or in combination, in response to local conditions (eg, bed management, PPE supplies), leading to all possible combinations of AS/CP de-implementation across the VA. Furthermore, VAMCs were able to re-implement these infection prevention practices alone or in combination as facility needs dictated throughout the pandemic response.

This “natural experiment” provided us an opportunity to understand the impact of AS, CPC, and CPI on their relative contributions toward preventing MRSA HAIs. In this study, we used comprehensive patient- and facility-level data from VAMCs to investigate the effects of changes in implementation of 3 major MRSA prevention practices on MRSA infection rates during the COVID-19 pandemic to inform future infection control policies in healthcare environments.

## METHODS

### Data Sources

The Multidrug-Resistant Organisms (MDRO) division of the VHA National Infectious Diseases Service (NIDS) assessed MRSA infection control policy at each of 123 VAMCs using an online electronic questionnaire with follow-up, ensuring complete data. Facilities were asked if they had stopped both AS and CP, stopped AS but continued CP, continued AS but stopped CP, or continued AS and CP. In each case, the survey clarified whether CP was continued/stopped for MRSA colonized patients, infected patients, or both. We used the results from this survey data collected between 1 July 2020 and 30 June 2022.

We extracted clinical data from VAs Corporate Data Warehouse (CDW) for all patients admitted to VAMCs during the same period. Patient data included date-time for admission, transfer and discharge, date-time, results, and sample source for both MRSA surveillance tests and all clinical cultures.

Our primary outcome was MRSA HAI rates at the facility-ward-month, defined as the monthly count of incident positive MRSA clinical cultures occurring any time during hospitalization from ≥3 days from admission through discharge in each facility and ward type (intensive care unit [ICU] versus medical/surgical wards [Med/Surg]), denominated by patient-days per month. An incident positive MRSA clinical culture is defined as a positive MRSA clinical culture with no prior positive MRSA clinical culture in the previous 365 days. Secondary outcomes were (1) 30-day postdischarge MRSA positive cultures to account for infections acquired during hospitalization but occurring after discharge and (2) HAIs restricted to MRSA-positive samples obtained from sterile body sites to maximize specificity and assess MRSA-positive results more likely to represent infection than colonization. Positive postdischarge cultures were attributed to the patient's ward location (ICU/non-ICU) at the time of discharge.

We also obtained the COVID-19 test results for all hospital admissions and considered patients infected at admission if they had a positive test in the 2 weeks prior to admission and up to 48 hours after admission. The burden of COVID-19 was defined as the proportion of admissions infected with COVID-19 and aggregated by facility-month and together with facility questionnaire responses on use of AS and CP for MRSA were included as time-varying covariates in our analyses. We also included facility-ward-month measures of clinical culture rate to account for facility-level differences in frequency of testing, where the clinical culture rate is defined as the number of clinical cultures obtained divided by the number of admissions.

Additional baseline covariates for our models included baseline facility-level burden of MRSA, census region, and facility complexity (a composite score generated by the VA to categorize VA facilities into 5 different complexity levels) [11]. The burden of MRSA was included using both admission prevalence and MRSA infection rate in the year prior to the COVID-19 pandemic (3/2019–3/2020). Admission prevalence was calculated as the proportion of MRSA surveillance tests taken upon admission in the year prior that were positive, and the infection rate was the number of incident infections divided by the number of patient-days at risk.

### Analysis

To explore whether Poisson variance assumptions were appropriate, the potential impact of small MRSA-positive counts for a subset of ward-facility-months, and possible site-specific clustering, we analyzed monthly MRSA HAI rates using 3 different regression approaches. Specifically, we used Poisson (Approach A) and Negative Binomial (Approach B) fixed effects regression and Poisson mixed-effects regression (Approach C). Poisson regression modeled counts of positive MRSA cultures offset by patient-days, while Negative Binomial extends Poisson to account for potential increased variation. Poisson mixed-effects regression was included to account for repeated measures and variation across units and was clustered within ward type and facility. For each regression approach, different analyses were run using 4 sets of nested covariates. The covariates represent metrics related to facility characteristics, important practices related to MRSA detection, historical burden of MRSA, and changes in COVID-19 burden at each facility. In combination, these covariates were used to evaluate the robustness of the results across a range of observable data and underlying assumptions, spanning models from the simplest (Model 1) to the most complex (Model 4).

The study was reviewed and approved by the University of Utah Institutional Review Board and the Research and Development Committee of the VA Salt Lake City Health Care System, which waived patient consent because the project relied on retrospective analysis of existing patient records. The analysis was implemented using the base package [12] from The R Project for Statistical Computing.

## RESULTS

### Summary

**Figure 1.**
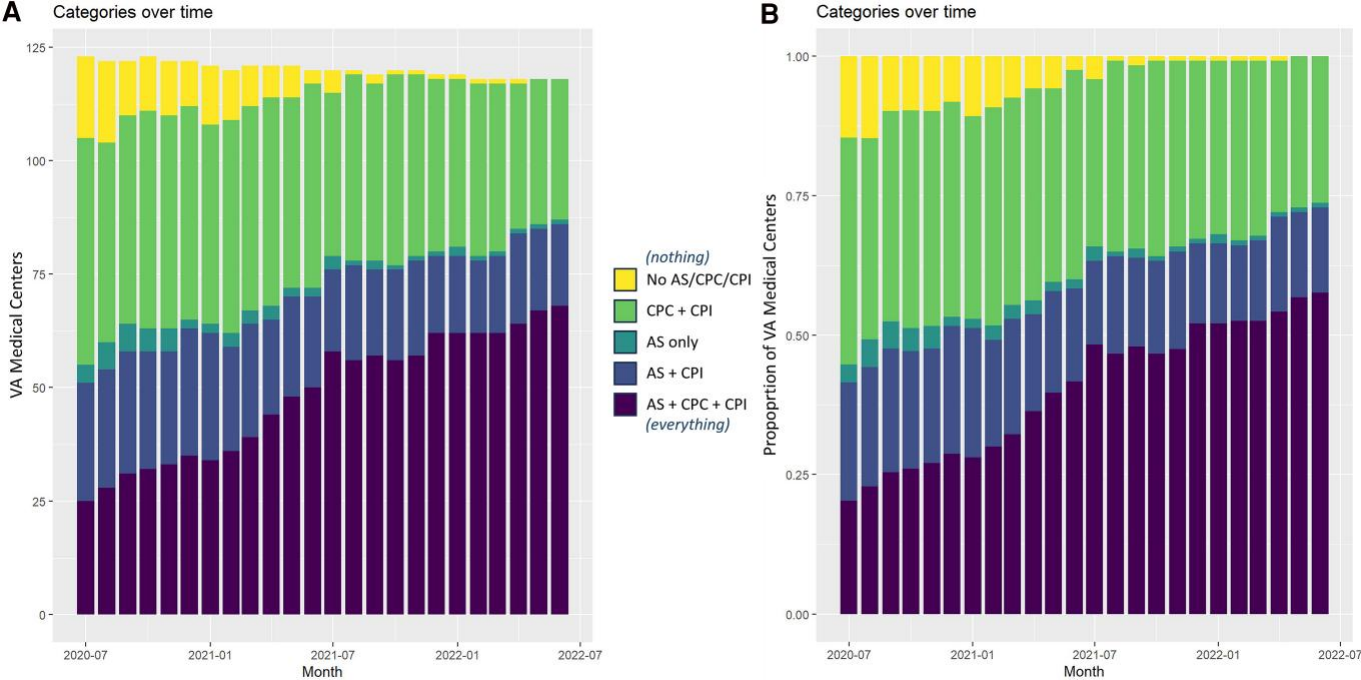
Number (*A*) and proportion (*B*) of VA acute care facilities endorsing use of active surveillance, contact precautions for MRSA-colonized, and contact precautions for MRSA-infected over time, stratified by combined infection prevention approach (*N* & 123 VA facilities), July 2020–July 2022. Abbreviations: AS, admission testing; CPC, contact precautions for MRSA-colonized; CPI, contact precautions for MRSA infection; MRSA, methicillin-resistant *Staphylococcus aureus.*

The VHA MDRO Office Prevention Division achieved participation from 100% of facilities in response to the questionnaire, and participation remained high for the duration of the study period (Figure 1, Panel A). Of the 123 facilities that provided survey data, 2 were excluded from this analysis for incomplete data on complexity score. There were 905 164 patients admitted to one of the remaining 121 VAMCs, with a total of 1708 positive MRSA cultures (385 non-sterile site, 584 sterile-site, 739 postdischarge) during the study period from 1 July 2020—30 June 2022. The median (IQR) of our primary and secondary outcomes was 0.49 (.45, .53) positive MRSA clinical cultures per 1000 patient-days, 0.16 (.14, .19) when restricted to sterile-site MRSA clinical cultures, and 0.70 (.64, .76) when including 30-day postdischarge cultures (Supplementary Figures 1–2).

**Figure 2.**
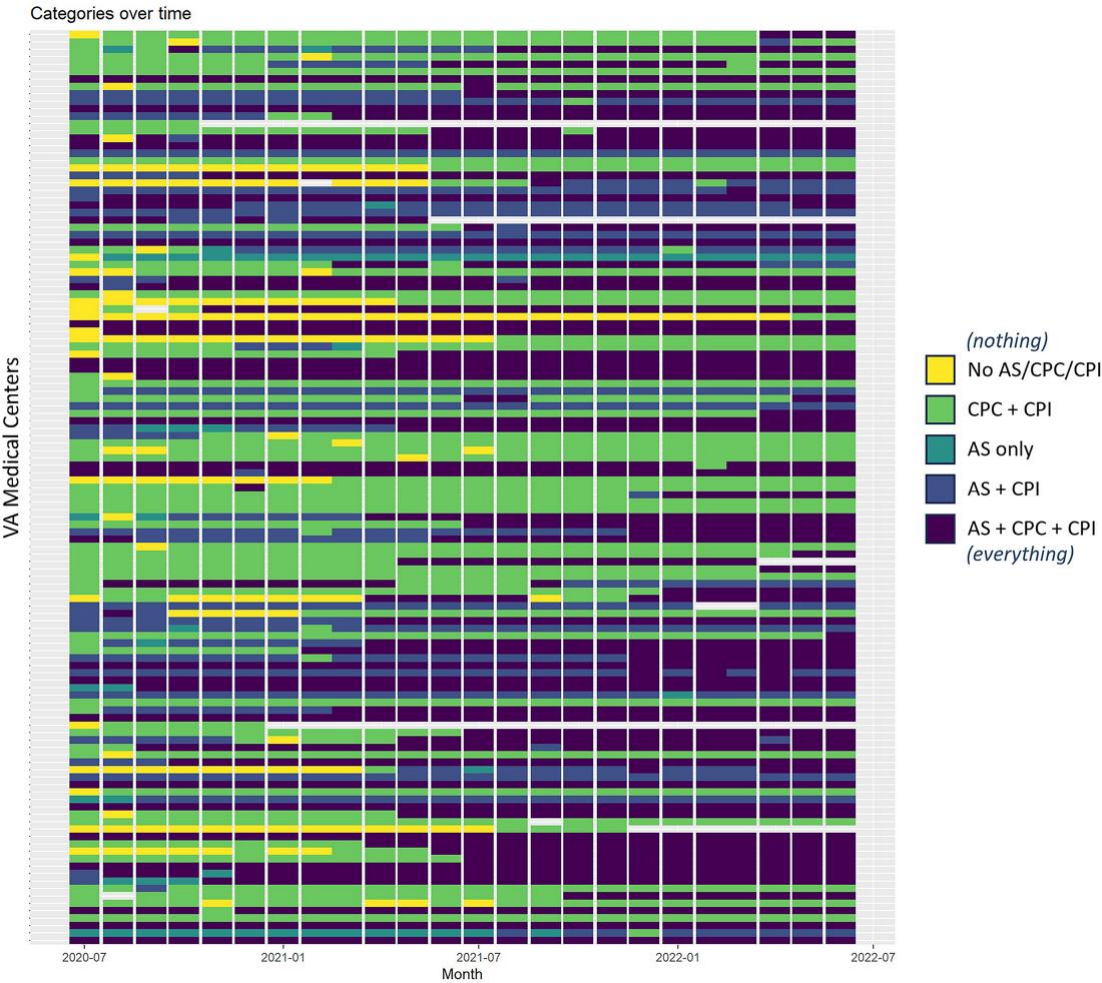
Frequency of changes in de-implementation and re-implementation of admissions testing, contact precautions for colonized patients, and contact precautions for patients with MRSA infections, stratified by acute care facility (N & 123 VA hospitals). Abbreviations: AS, admission testing; CPC, contact precautions for MRSA-colonized; CPI, contact precautions for MRSA infection; MRSA, methicillin-resistant *Staphylococcus aureus.*

In the first month of the survey, July 2020, ∼20% of facilities indicated that they had elected to continue all elements of the MRSA prevention initiative (Figure 1, Panel B); however, by the end of the study period in June 2022, that percentage increased to 57%. More than 10% of facilities elected to discontinue all MRSA prevention practices early on in the pandemic, but by the mid-point of the study period, nearly all facilities (95%) were engaged in at least one practice. Throughout the study, a majority of facilities continued CPC and CPI, though many of those facilities indicated that they discontinued AS. On average, fewer than 3 facilities per month (range, 1–6) elected only to continue AS without CPC or CPI, although this number declined dramatically with only 1 facility continuing to implement AS alone during 9 of the last 12 months in the study. A majority of facilities maintained consistent practices throughout the study period, changing practices no more than once (51% of facilities) or twice (70%) during the study period (Figure 2).

### Approach A: Poisson Regression

Using Poisson regression, we found with the simplest model (Model 1) that MRSA infection rates significantly increased when facilities either discontinued all 3 infection prevention practices or discontinued CPC while continuing to use AS and CPI. The overall culture rate was associated with increased MRSA infection rates; however, MRSA infection rates were lower on Med/Surg wards than on ICUs (Table 1, Model 1). After accounting for the baseline MRSA infection rate and admission prevalence, discontinuation of AS, CPC, and CPI were no longer associated with increases in MRSA infection rates (Table 1, Models 2–4). With the addition of facility covariates, we still found that the overall culture rate as well as combined use of both AS and CPI, but not CPC, were associated with increased MRSA rates.

**Table 1.** Association of AS, CPI, and CPC Infection Prevention Practices With MRSA Infection Rate, Adjusted for Patient- and Facility-level Characteristics From the Poisson Regression

Poisson				
	Model 1	Model 2	Model 3	Model 4
Intercept	**−7.03** *** **(0.05)**	**−7.06** *** **(0.05)**	**−7.12** *** **(0.07)**	**−7.13** *** **(0.07)**
AS + CPI + CPC	…	…	…	…
AS + CPI	0.16** (0.06)	0.13* (0.06)	0.15* (0.06)	0.15* (0.06)
AS only	**0.03** (0.13)	**−0.03** (0.13)	**0.13** (0.14)	**0.14** (0.14)
CPC + CPI	**0.07** (0.05)	**0.07** (0.05)	**0.06** (0.05)	**0.06** (0.05)
No AS, CPC or CPI	**0.39** *** (0.09)	**0.16** (0.09)	**0.13** (0.10)	**0.13** (0.10)
Med/Surg	**−0.90** *** (0.04)	**−0.90** *** (0.04)	**−0.90** *** (0.04)	**−0.89** *** (0.05)
Culture Rate	**0.14** *** (0.03)	**0.11** *** (0.03)	**0.11** *** (0.03)	**0.11** *** (0.03)
Time (months)	**−0.01** (0.02)	**−0.02** (0.02)	**−0.02** (0.02)	**−0.02** (0.02)
Admission Prevalence (year before 3/2020)	…	0.07* (0.03)	0.07* (0.03)	0.07* (0.03)
Infection Rate (year before 3/2020)	…	**0.27** *** (0.03)	**0.25** *** (0.03)	**0.24** *** (0.03)

^***^ *P* < .001; **P < .01; *P < .05.

Regression coefficients are bolded and associated standard errors are in parentheses.

### Approach B: Negative Binomial Regression

The results from our analyses when using Negative Binomial regression revealed the same pattern of associations as those obtained from our Poisson regression analysis (Supplementary 2—supplementary material).

### Approach C: Mixed-effects Poisson Regression

In the Poisson mixed-effects model, we also found that both ward type and the overall rate of culturing are associated with MRSA infection rates (Table 2, Model 1), and inclusion of facility-level MRSA infection rate in the year prior was also associated with increased rates of MRSA (Table 2, Models 2–4). In contrast to the fixed-effects regression analyses, we find that facility-level MRSA prevention practices (AS/CPC/CPI) are not significantly associated with MRSA infection rates in any of the models.

**Table 2.** Association of AS, CPI, and CPC Infection Prevention Practices With MRSA Infection Rate, Adjusted for Patient- and Facility-level Characteristics From the Poisson Mixed-effects Regression Clustered by Ward Type (ICU vs Med/Surg) and Facility

Poisson Mixed-Effects				
	Model 1	Model 2	Model 3	Model 4
Intercept	**−7.07** *** **(0.07)**	**−7.08** *** **(0.07)**	**−7.19** *** **(0.11)**	**−7.20** *** **(0.11)**
AS + CPI + CPC	…	…	…	…
AS + CPI	**0.02 (0.08)**	**0.03 (0.08)**	**0.05 (0.08)**	**0.05 (0.08)**
AS only	**0.08 (0.19)**	**0.06 (0.18)**	**0.15 (0.18)**	**0.15 (0.18)**
CPC + CPI	**0.05 (0.08)**	**0.03 (0.07)**	**0.03 (0.07)**	**0.03 (0.07)**
No AS, CPC or CPI	**0.16 (0.13)**	**0.13 (0.12)**	**0.12 (0.12)**	**0.12 (0.12)**
Med/Surg	**−0.91** *** **(0.06)**	**−0.91** *** **(0.06)**	**−0.91** *** **(0.06)**	**−0.90** *** **(0.06)**
Culture Rate	**0.15** *** **(0.03)**	**0.14** *** **(0.03)**	**0.14** *** **(0.04)**	**0.14** *** (**0.04)**
Time (months)	**−0.02 (0.02)**	**−0.02 (0.02)**	**−0.02 (0.02)**	**−0.03 (0.02)**
Admission Prevalence (year before 3/2020)	**…**	0.08* **(0.04)**	**0.08 (0.04)**	**0.07 (0.04)**
Infection Rate (year before 3/2020)	**…**	**0.22** *** **(0.05)**	**0.18** *** **(0.05)**	**0.18** *** **(0.05)**
Number of groups Ward Type: Facility	**228**	**228**	**228**	**228**
Number of groups Facility	**121**	**121**	**121**	**121**
Variance Ward Type: Facility (Intercept)	**0.04**	**0.04**	**0.04**	**0.04**
Variance Facility (Intercept)	**0.13**	**0.08**	**0.06**	**0**.**06**

^***^ *P* < .001; *P < .05.

Regression coefficients are bolded and associated standard errors are in parentheses. The remaining entries provide common metrics related to mixed-effects regression.

### Secondary Outcomes

When both restricting our analysis to sterile-site cultures and inclusion of 30-day postdischarge cultures, we see similar results across the different regression approaches and modeled covariates as was observed in our primary analysis (Supplementary 4—9).

### Model AICs

The AIC for the different models (Table 3) indicates that the models tend to have a better fit with increasing numbers of covariates and as we relax model assumptions across the differing regression approaches. In particular, the covariates included in Model 1 have a distinctly poor model fit compared to each of the other models with additional covariates, and the Poisson mixed-effects regression model has the best performance with very similar AICs using Models 2–4.

**Table 3.** Akaike Information Criterion for Each Model and Regression Approach Incorporated into our Analysis Providing Metric of Model Fit

Akaike Information Criterion			
	Poisson	Negative-binomial	Poisson Mixed-effects
Model 1	8387.20	8361.87	8223.38
Model 2	8278.46	8267.31	8203.64
Model 3	8266.59	8256.13	8202.31
Model 4	8265.64	8255.57	8201.94

## DISCUSSION

The findings from our study explore the potential impacts of 3 common MRSA infection prevention practices by facility and month within a large, integrated healthcare system. The temporary suspension and subsequent variable reimplementation of MRSA AS and CPs allowed us to assess the effectiveness of these prevention measures and the robustness of these findings across a range of assumptions. Our results suggest that in the simplest models, discontinuation of AS, CPC, and CPI was associated with increased MRSA infection rates, consistent with prior findings [6]. However, inclusion of the baseline MRSA infection rate and admission prevalence substantially attenuated the effect estimates for MRSA precautions, which provide evidence of these baseline covariates being important confounding variables that must be accounted for to accurately assess the impact of MRSA prevention practices.

Many facilities initially discontinued one or more MRSA prevention practices but the majority gradually reinstated these measures. By the end of the study period, more than half of the facilities had reinstated all 3 of these components of the MRSA Prevention Initiative. This suggests that facilities perceived the importance of the 3 infection control strategies even as pandemic-related constraints eased. The observed trend where facilities were more likely to continue CPC and CPI but not AS may reflect logistical challenges in conducting surveillance testing during the pandemic.

One key finding of our study is the variation in results across different modeling approaches. The Poisson and Negative Binomial regression models demonstrated that discontinuation of AS, CPC, and CPI was associated with increased MRSA infection rates, particularly when culture rates were high. However, our Poisson mixed-effects models did not find a strong association between facility-level MRSA prevention practices and infection rates after accounting for prior facility MRSA burden. This discrepancy underscores the importance of understanding the sources of variation across models. Differences in how each approach accounts for unobservable facility-level factors, clustering, and overdispersion may explain these divergent results. The challenges of statistical interpretation highlight the need for careful consideration of modeling assumptions in infection control research, particularly when those analyses are used to influence policymaking. Our secondary analyses, which restricted outcomes to sterile-site cultures and included postdischarge infections, corroborated the primary findings in each of the models. This robustness across different analytical approaches underscores the reliability of our results and indicates that variations in MRSA prevention measures may not have measurable consequences on infection rates.

The differences in performance, as indicated by AIC values, further highlight the complexity of MRSA transmission dynamics. The Poisson mixed-effects model, which is generally preferred when observations are clustered or non-independent, provided a better fit than the fixed-effects models, and inclusion of key covariates led to better fit, reinforcing the importance of considering facility-specific factors when evaluating infection prevention strategies. However, understanding why different modeling approaches lead to different conclusions remains an open question that warrants further investigation.

While our study of AS, CPC, and CPI was conducted within a pandemic response setting, the deimplementation of one or more MRSA infection prevention strategies in endemic settings has been a subject of increasing debate [13–16]. Previous work has focused on pre-post studies examining the effect of discontinuing CPC and/or CPI on infection rates and has reported no change in hospital infection rates [3, 17–20]. However, many of these findings have primarily been single institution changes in infection prevention policy and conducted over a shorter follow-up time than was possible with our analysis. These studies also often have concurrent, intentional increased emphasis on horizontal infection prevention measures; such bundling may obscure the changes in MRSA infection rates that may otherwise occur with CPC and CPI deimplementation. Our study also had the ability to compare MRSA infection rates in facilities that continued using these MRSA prevention practices as well as facilities that readopted some or all MRSA Prevention Initiative strategies.

Beyond statistical considerations, the broader implications of COVID-19-related infection control changes on MRSA HAIs remain unresolved. The time scales used in our analysis may not fully account for delayed impacts of MRSA prevention practice changes, raising further questions about how best to detect changes in infection rates over time. Additionally, although this study was done across a large healthcare system, it is possible that more facilities or longer follow-up are needed in order to detect any changes in MRSA infection rates, as it has been shown that many intervention studies tend to be underpowered [21]. Future modeling and simulation work could help to clarify these questions. The long-term impact of withdrawing components of the MRSA prevention bundle remains an open and unanswered question that requires continued monitoring and investigation.

### Limitations

While our study had several notable strengths and we were able to adjust for known facility-level confounders, unmeasured factors such as staffing shortages, variations in adherence to infection control protocols, and differences in patient case mix may have influenced MRSA rates. Additionally, the widespread use of personal protective equipment (PPE), including gowns and gloves for COVID-19 patients, may have indirectly affected MRSA transmission dynamics in ways not captured by our models and remain to be investigated.

Another limitation is the fact that we are not able to truly differentiate clinical infections from colonization relying solely on clinical culture data. While we attempted to use a surrogate through restricting to sterile-site cultures, we may still have missed infections that were not captured in clinical cultures. Furthermore, attributing postdischarge infections to the patient's last inpatient ward assumes a direct link between hospitalization and subsequent infection, which may not always be accurate.

Lastly, uncertainty about the relationship between the timing of policy change and the corresponding response in practice (including the implementation rate and compliance of practice change) limits our ability to closely link changes in infection control practices to outcomes.

## CONCLUSIONS

Our study provides valuable insights into the relationship between MRSA infection prevention practices and infection rates and informs the ongoing discussion on effective infection prevention strategies to prevent MRSA transmission within acute healthcare settings. Our findings suggest that in the simplest model, discontinuation of AS, CPC, and CPI was associated with increased MRSA rates, but this association was mitigated when accounting for facility-level MRSA burden prior to the pandemic. These results suggest that the effectiveness of MRSA prevention measures is highly context-dependent, emphasizing the need for policies that adapt to facility-specific conditions and resource constraints.

Future research should explore the long-term impacts of these policy changes, particularly in the context of evolving healthcare challenges. Additionally, further investigation into the role of facility-specific factors in shaping infection control outcomes could provide deeper insights into optimizing MRSA prevention strategies in hospital settings. Moreover, understanding the effects of different modeling approaches on infection control research remains critical, particularly in addressing questions about unobservable data, facility-level variation, and appropriate time scales for detecting changes in infection rates and when it comes to setting and evaluating policy.

## Supplementary Material

ciag176_Supplementary_Data

## Supplementary Data

Supplementary materials are available at *Clinical Infectious Diseases* online. Consisting of data provided by the authors to benefit the reader, the posted materials are not copyedited and are the sole responsibility of the authors, so questions or comments should be addressed to the corresponding author.
