# Supplementary material for: Estimating Changes in Facility Methicillin-Resistant Staphylococcus aureus (MRSA) Infection Rates Due to Changes in MRSA Precaution Policy
Source: Clin Infect Dis. 2026 Mar 13;83(1):e113–9. doi: 10.1093/cid/ciag176 (PMC13393110; doi:10.1093/cid/ciag176)
Supplement: ciag176_Supplementary_Data [file ciag176_supplementary_data.docx]

**Supplementary Material**

Below, we present the complete model results from our original analysis based on our primary outcome of incident positive MRSA clinical cultures occurring any time during hospitalization from ≥ 3 days from admission through discharge, denominated by patient-days per month. We also include our secondary outcomes: 1) HAIs restricted to MRSA-positive samples obtained from sterile body sites, and 2) 30-day post-discharge MRSA positive cultures to account for infections acquired during hospitalization but occurring after discharge.

**Incident Positive MRSA clinical cultures**

**S1.** Association of AS, CPI, and CPC infection prevention practices with MRSA infection rate, adjusted for patient- and facility-level characteristics from Poisson regression.

**Poisson regression**


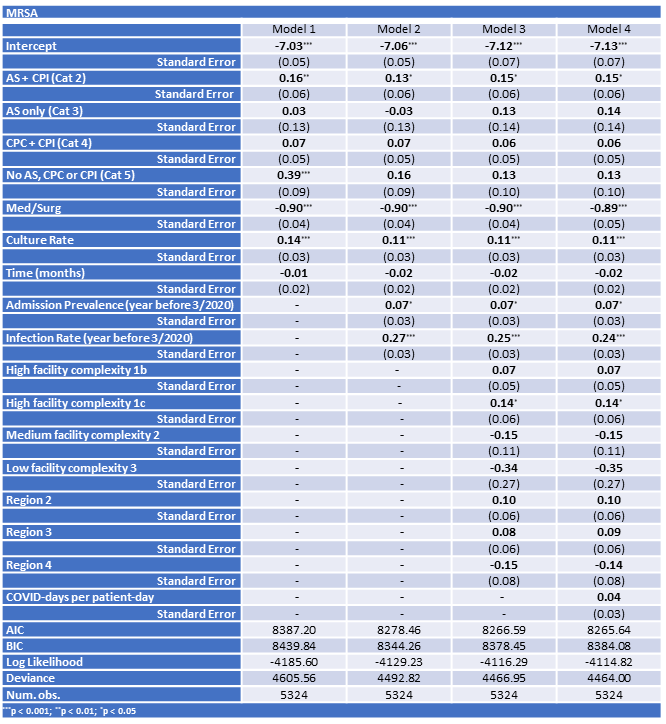


*Note:* Regression coefficients are bolded and associated standard errors are in parentheses.

**S2.** Association of AS, CPI, and CPC infection prevention practices with MRSA infection rate, adjusted for patient- and facility-level characteristics from Negative binomial regression.

**Negative binomial regression**


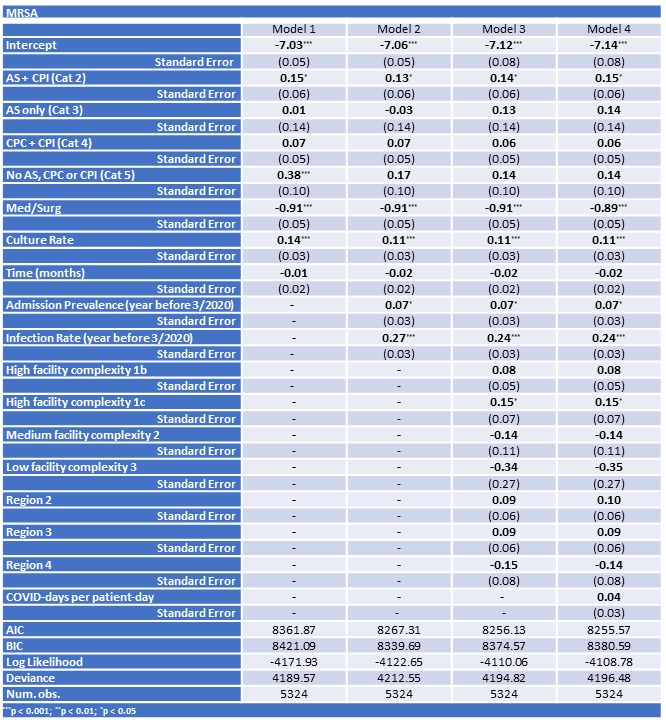


*Note:* Regression coefficients are bolded and associated standard errors are in parentheses.

**S3.** Association of AS, CPI, and CPC infection prevention practices with MRSA infection rate, adjusted for patient- and facility-level characteristics from mixed-effects Poisson regression clustered by WardType (ICU vs Med/Surg) and Facility (Sta6a).

**Mixed-effects Poisson regression**


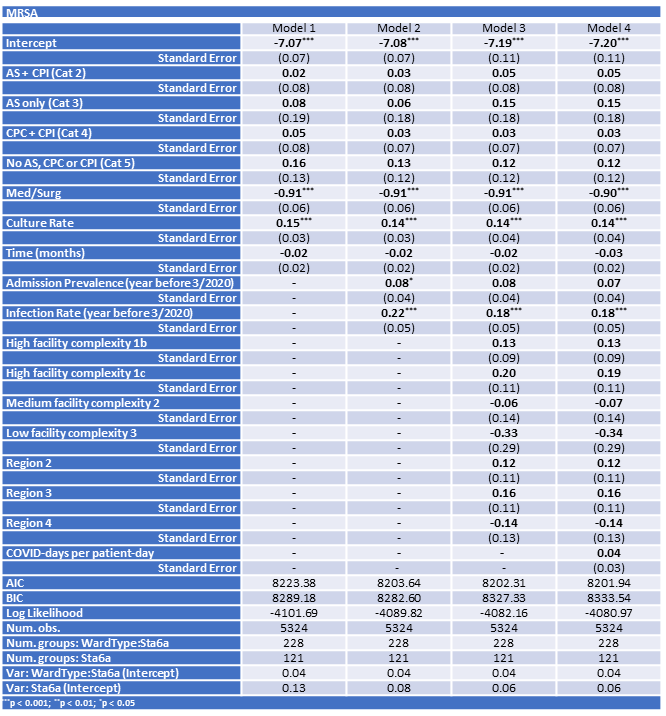


*Note*: Regression coefficients are bolded and associated standard errors are in parentheses. The remaining entries provide common metrics related to mixed-effects regression.

**Incident Positive MRSA Sterile-site clinical cultures**

**S4.** Association of AS, CPI, and CPC infection prevention practices with MRSA infection rate, adjusted for patient- and facility-level characteristics from Poisson regression.

**Poisson regression**


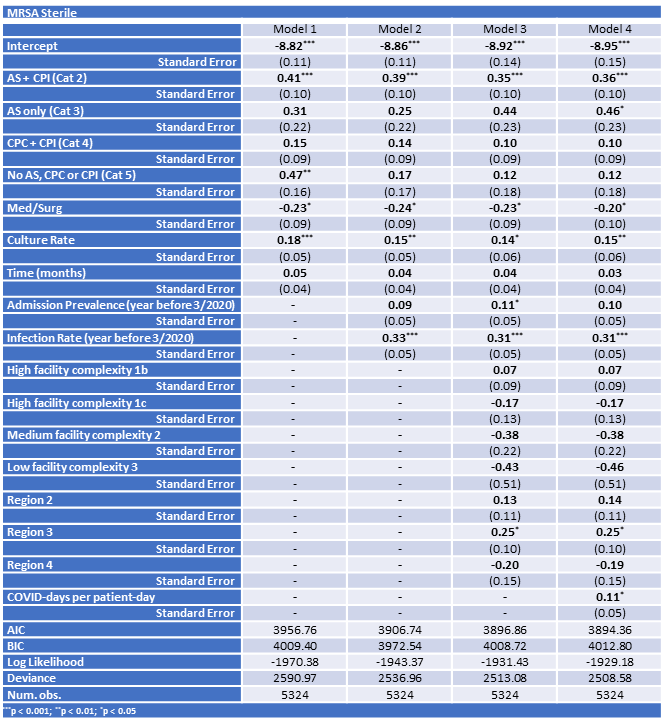


*Note:* Regression coefficients are bolded and associated standard errors are in parentheses.

**S5.** Association of AS, CPI, and CPC infection prevention practices with MRSA infection rate, adjusted for patient- and facility-level characteristics from Negative binomial regression.

**Negative binomial regression**


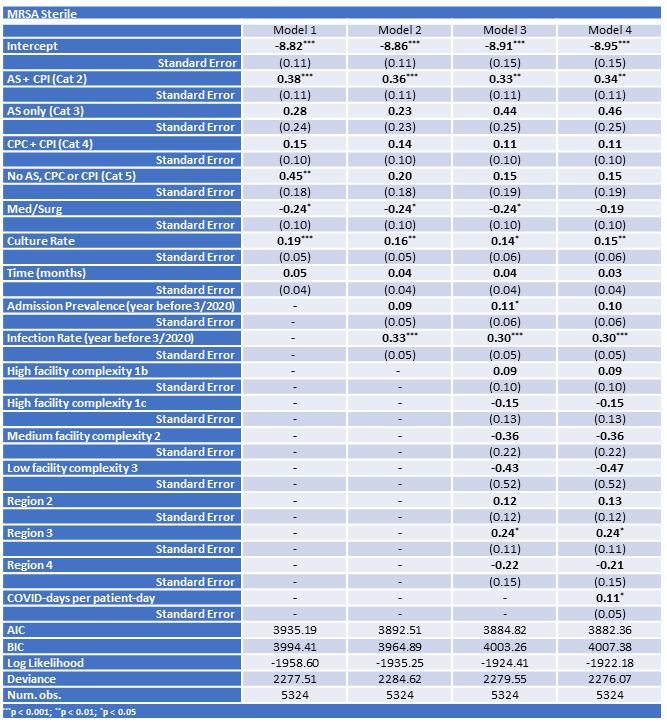


*Note:* Regression coefficients are bolded and associated standard errors are in parentheses.

**S6.** Association of AS, CPI, and CPC infection prevention practices with MRSA infection rate, adjusted for patient- and facility-level characteristics from mixed-effects Poisson regression clustered by WardType (ICU vs Med/Surg) and Facility (Sta6a).

**Mixed-effects Poisson regression**


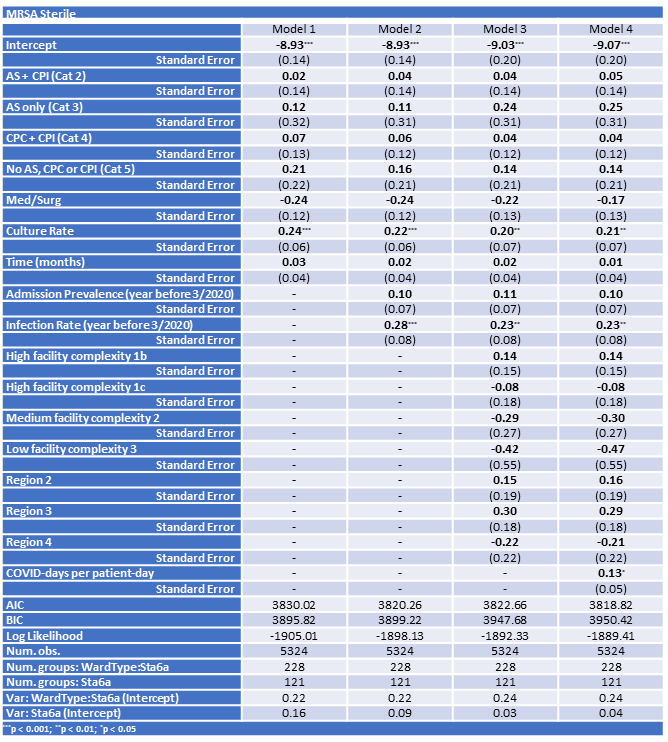


*Note*: Regression coefficients are bolded and associated standard errors are in parentheses. The remaining entries provide common metrics related to mixed-effects regression.

**30-day Post Discharge Analysis**

**S7.** Association of AS, CPI, and CPC infection prevention practices with MRSA infection rate, adjusted for patient- and facility-level characteristics from the Poisson regression.

**Poisson regression**


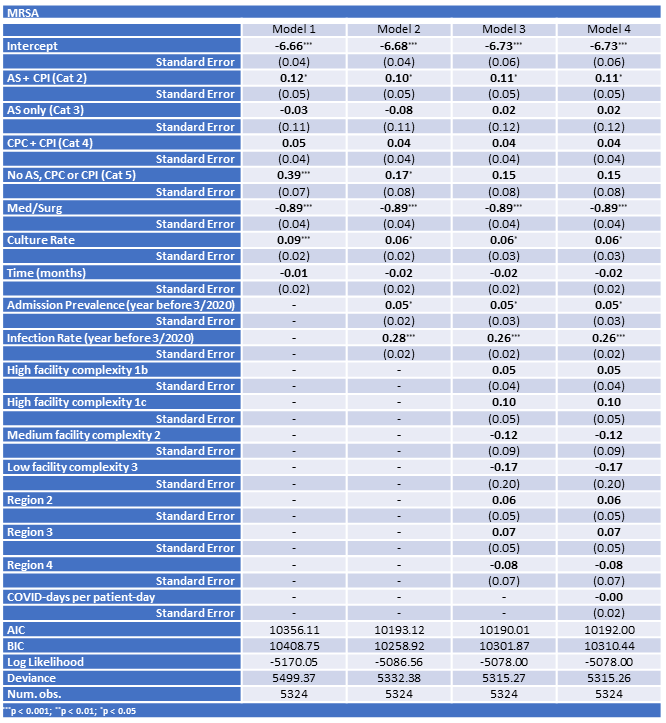


*Note:* Regression coefficients are bolded and associated standard errors are in parentheses.

**S8.** Association of AS, CPI, and CPC infection prevention practices with MRSA infection rate, adjusted for patient- and facility-level characteristics from Negative binomial regression.

**Negative binomial regression**


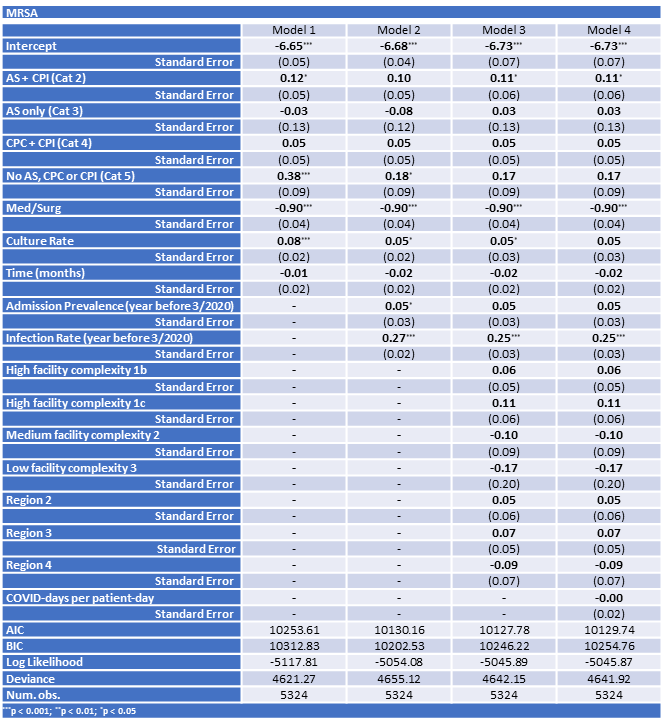


*Note:* Regression coefficients are bolded and associated standard errors are in parentheses.

**S9.** Association of AS, CPI, and CPC infection prevention practices with MRSA infection rate, adjusted for patient- and facility-level characteristics from mixed-effects Poisson regression clustered by WardType (ICU vs Med/Surg) and Facility (Sta6a).

**Mixed-effects Poisson regression**


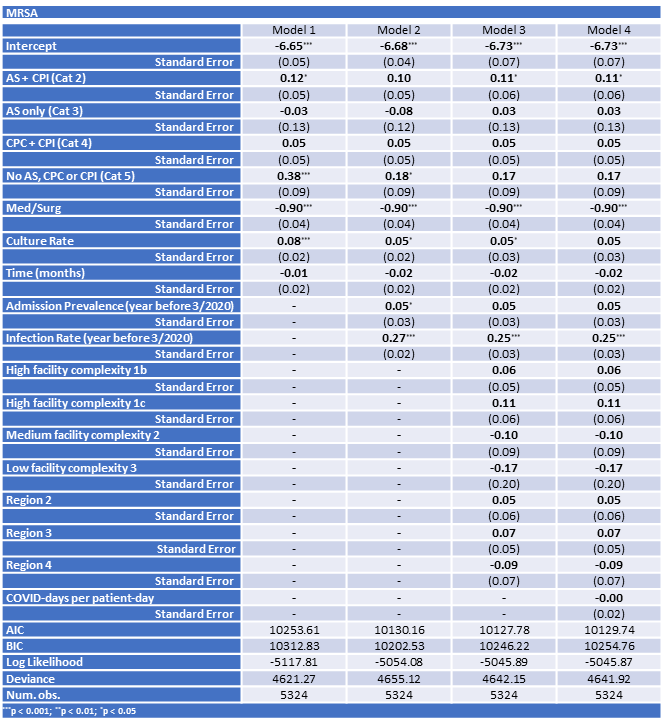


*Note*: Regression coefficients are bolded and associated standard errors are in parentheses. The remaining entries provide common metrics related to mixed-effects regression.

**MRSA Rates and Variation**

In this section, we include figures illustrating the temporal variation in our model outcomes to provide context related to the outcomes we are modeling in this study.


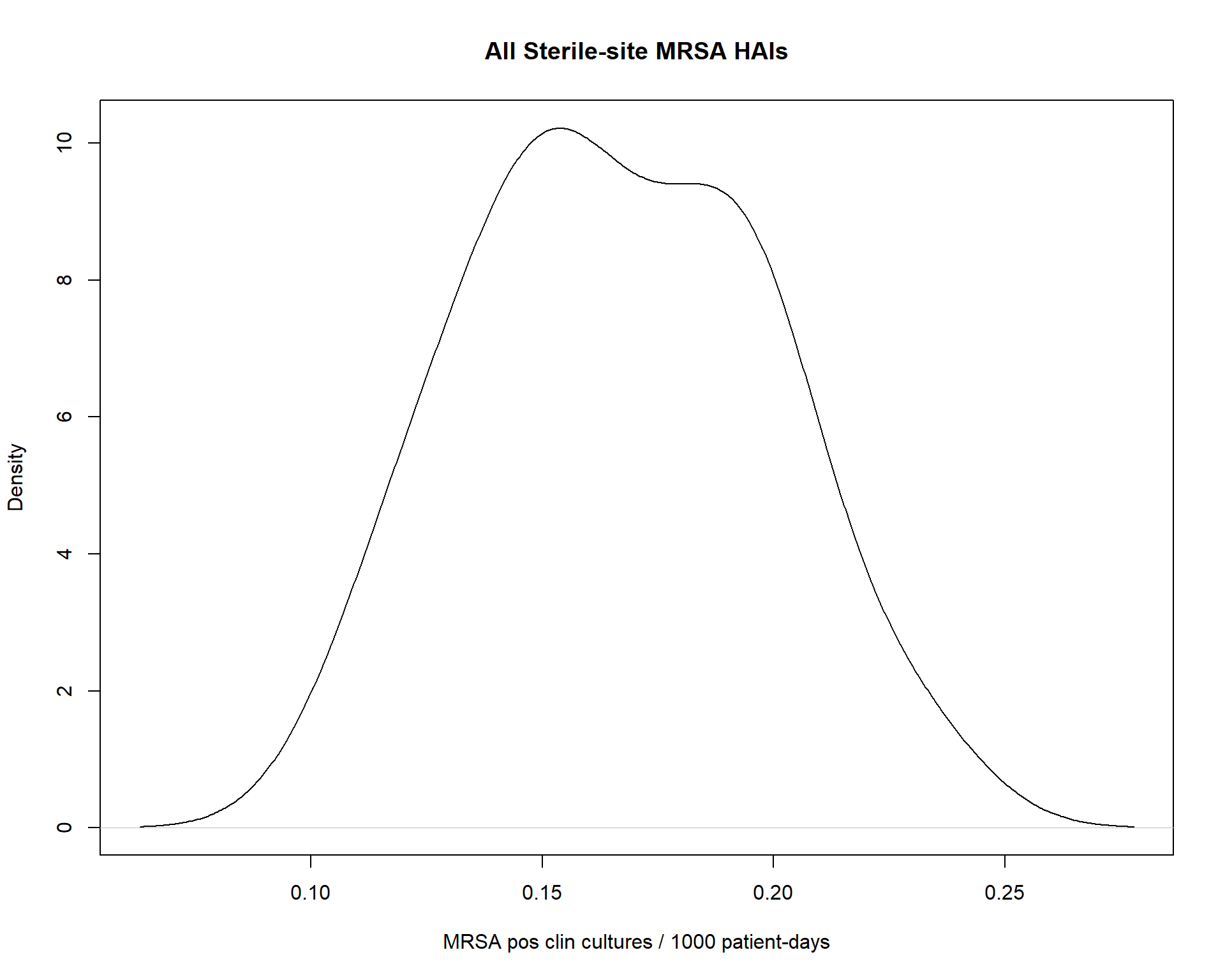

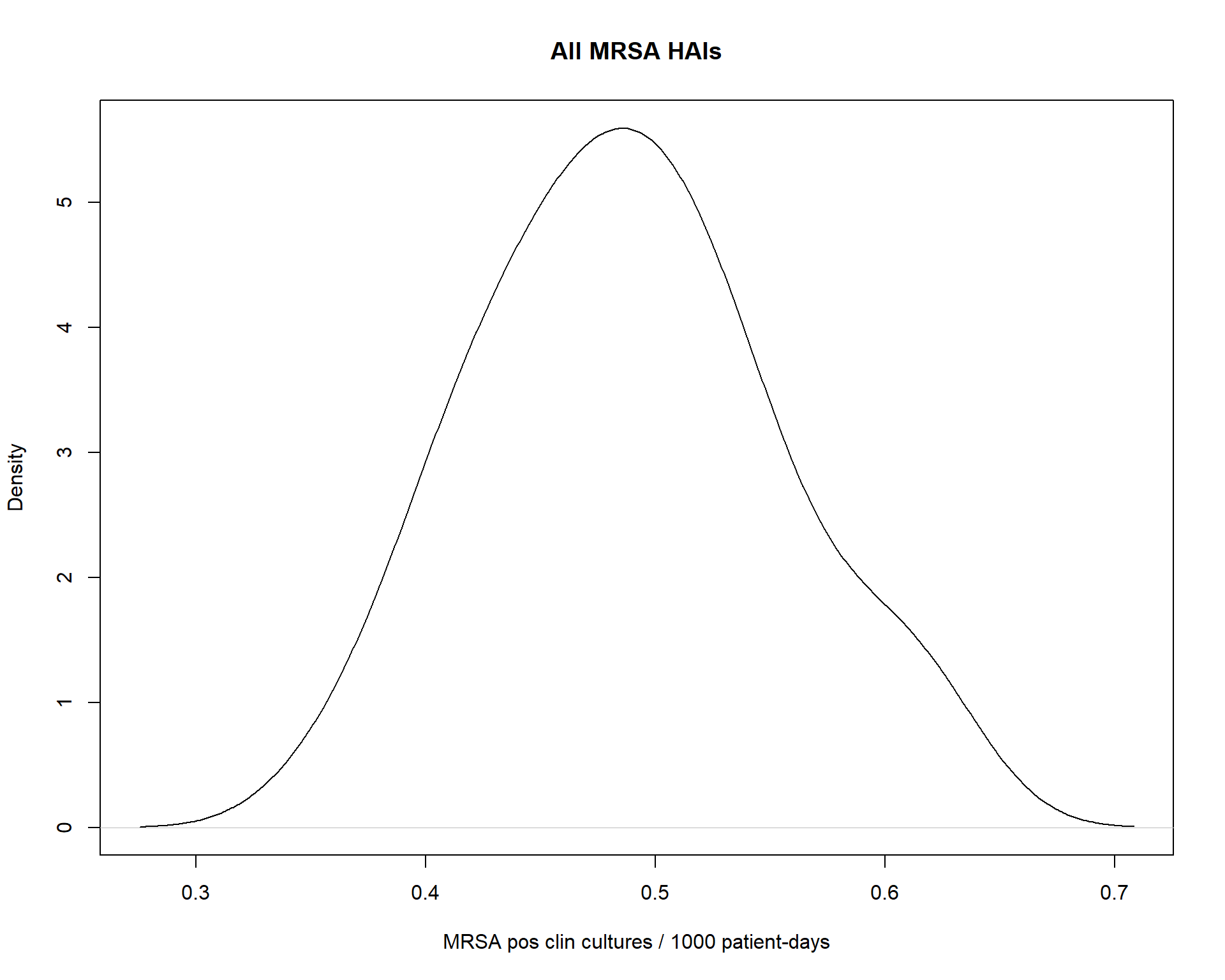


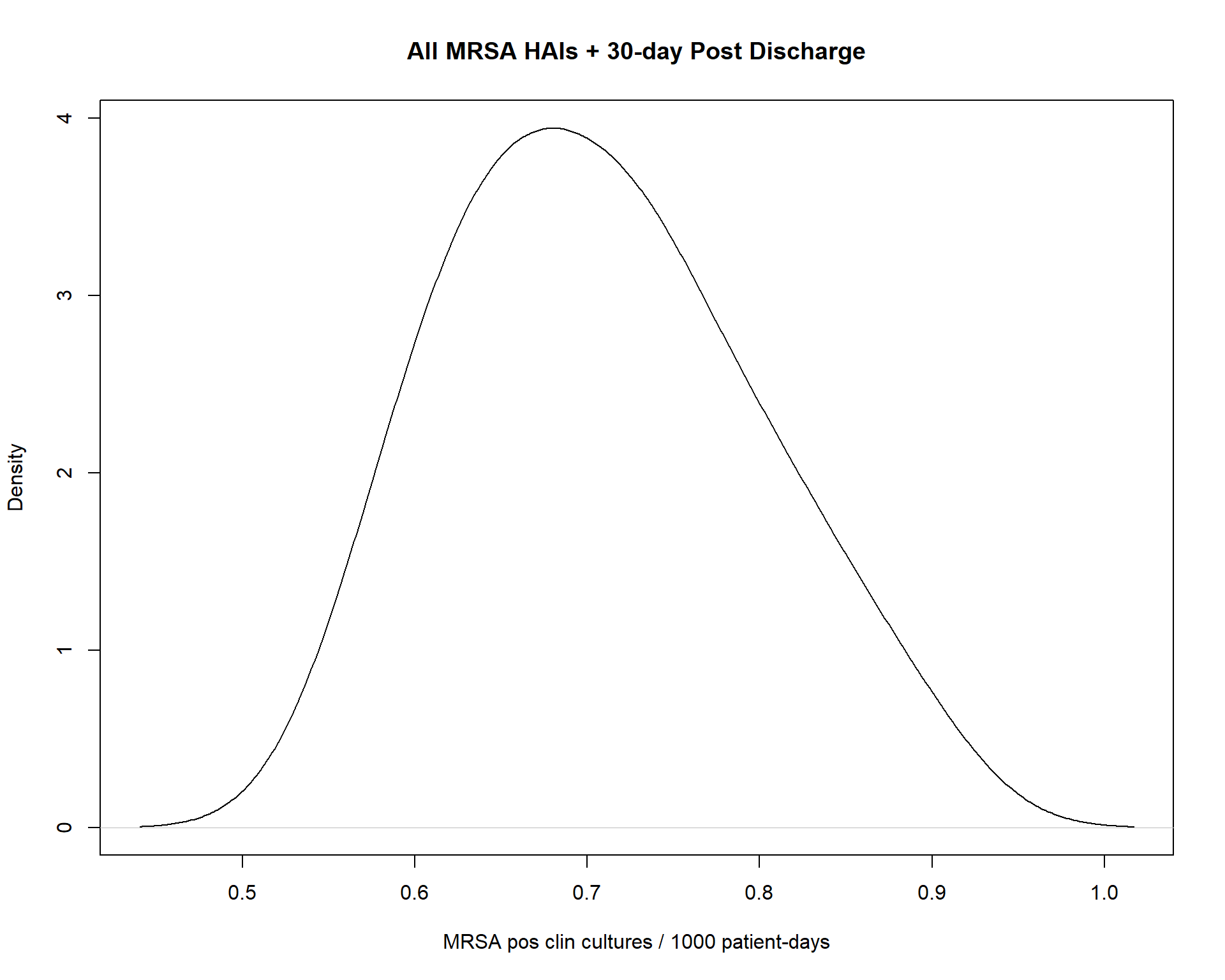


**SFigure 1.** Density plot showing the distribution of our monthly primary and secondary outcomes across the study period.


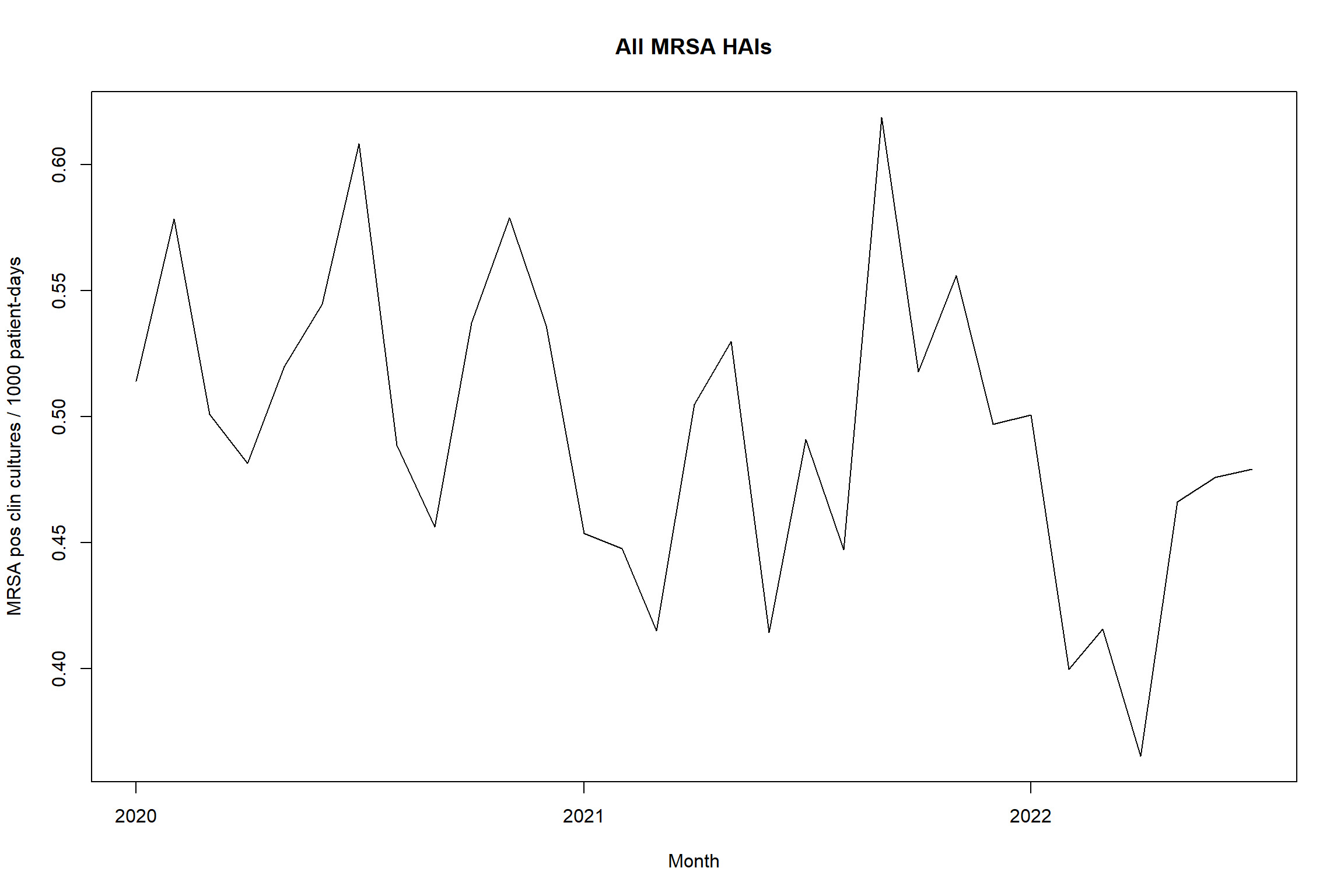

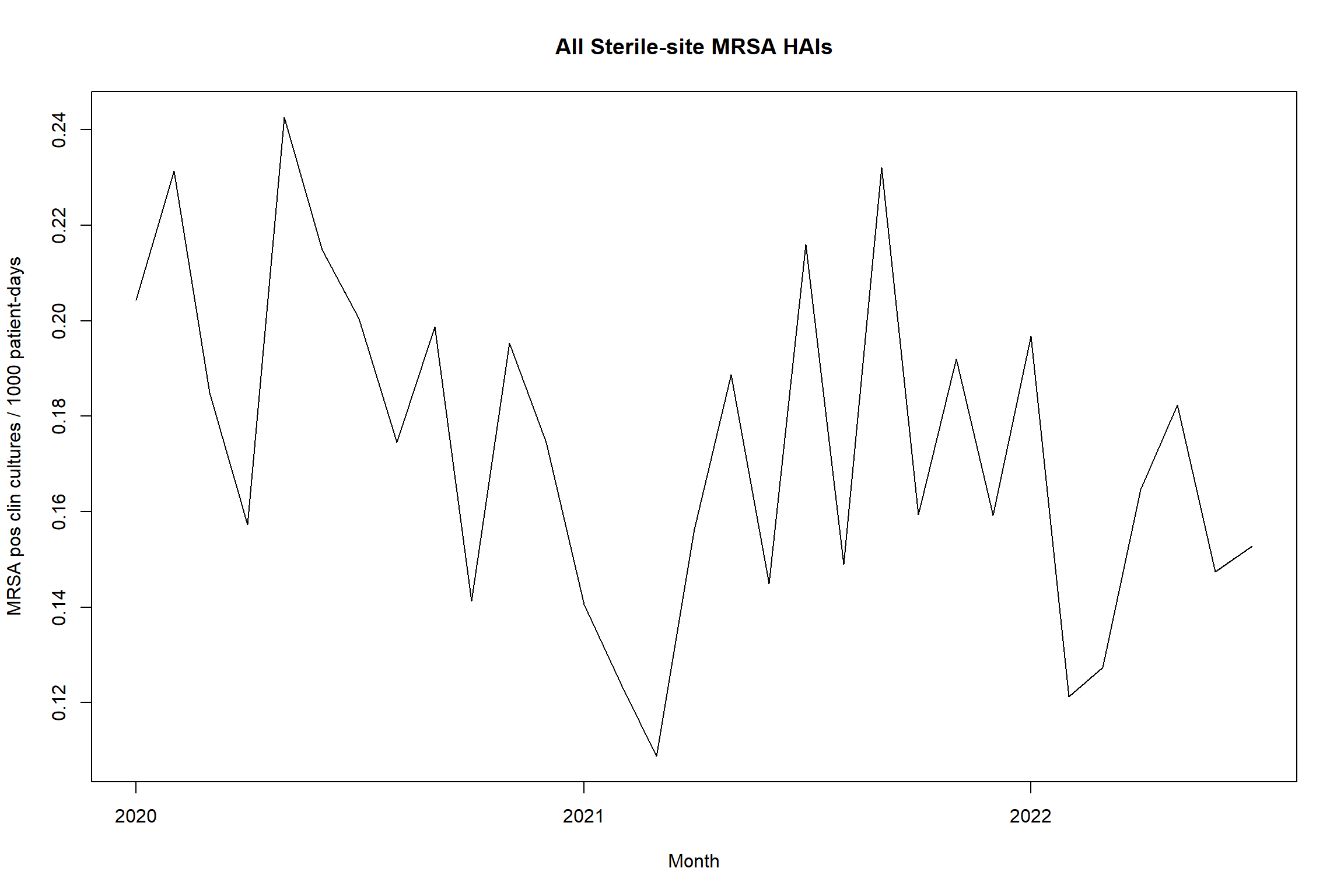

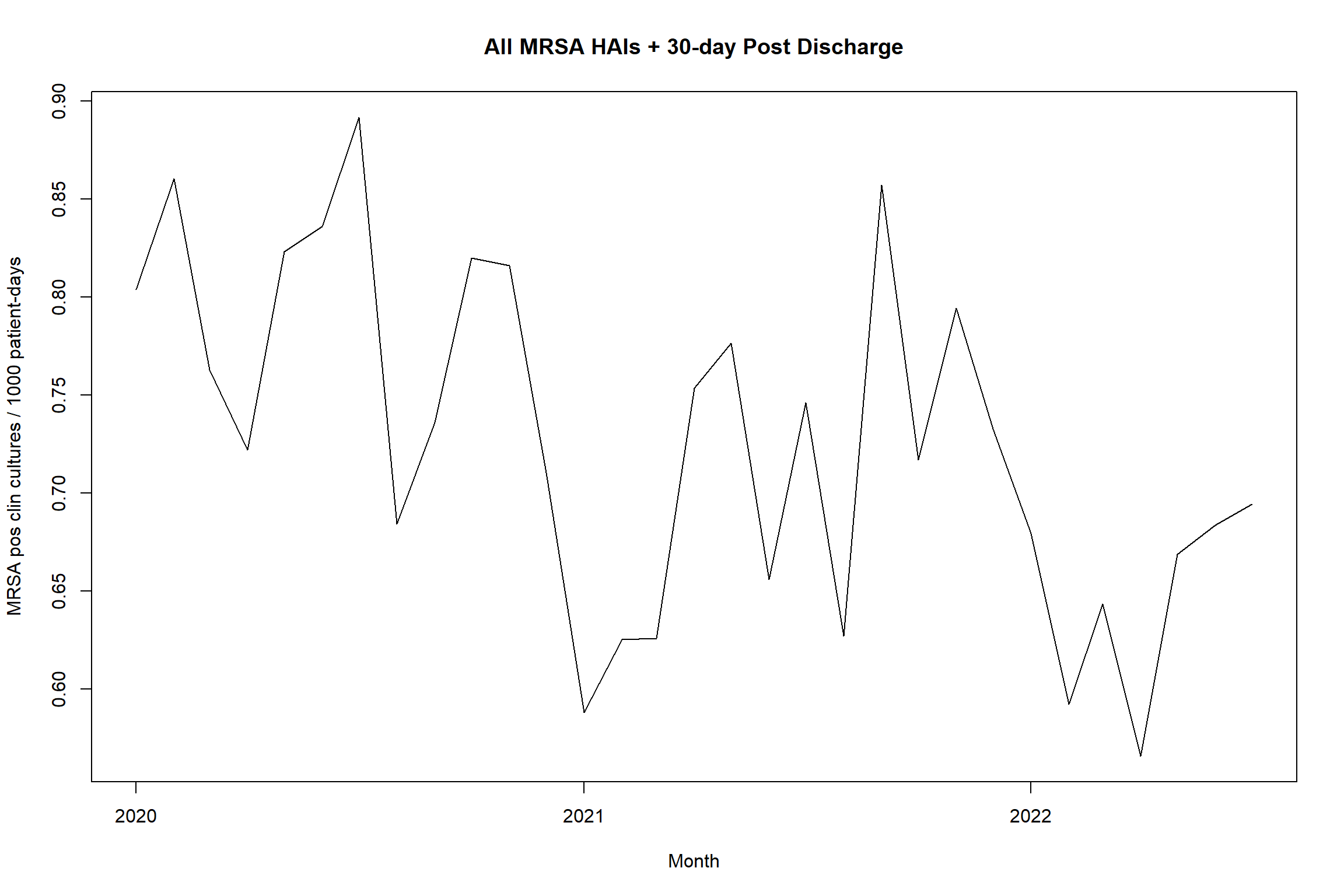


**SFigure 2.** Plot showing the monthly temporal variation of our monthly primary and secondary outcomes over time.
